# The Brazilian Contributions to Congenital Cardiac Surgery

**DOI:** 10.21470/1678-9741-2020-0600

**Published:** 2020

**Authors:** Elizabeth H. Stephens, Joseph A. Dearani

**Affiliations:** 1 Department of Cardiovascular Surgery, Mayo Clinic, Rochester, MN, United States of America

The world of congenital cardiac surgery has been profoundly impacted by surgeons heralding from Brazil. These contributions date back to the infancy of congenital cardiac surgery and continue today. In this editorial, we briefly reflect on the legacy of the contributions to congenital cardiac surgery from Brazil, highlighting some of the key pioneers and their role in shaping the specialty.

## EURYCLIDES DE JESUS ZERBINI

Born in 1912, in the rural countryside, the son of an Italian elementary school teacher, Zerbini pursued medicine as a result of pressure from his father^[1]^. While at São Paulo’s Santa Casa de Misericórdia, a fashionable teaching hospital, he met the famous surgeon Alípio Correa Netto, who became Zerbini’s mentor and guide throughout his career^[1]^. Zerbini practiced thoracic surgery in the 1930s, consisting mostly of tuberculosis treatment, but in 1942 he performed his first cardiac surgery on a six-year-old child with cardiac tamponade from a fragment of an anvil. The shard had injured the left anterior descending coronary artery and this vessel was ligated, saving the boy’s life^[1]^. After touring the United States of America and observing surgical legends, such as Drs. Blalock and Lillehei, Zerbini brought cardiac surgery back to Brazil, performing the Blalock-Taussig shunt, ligation of the patent arterial duct, and coarctation repair^[1]^. Zerbini then devoted much of his attention to tetralogy of Fallot, performing 480 repairs and presenting his analysis of 103 of his initial repairs in 1968^[1]^. Zerbini became known as an international expert in this area. Constantly innovating, Zerbini and colleagues developed a homologous dura mater prosthetic valve in 1971, which was used extensively throughout the country and abroad, but it was discontinued because of structural defects^[1]^. While Christian Barnard famously performed the first human heart transplant on December 4, 1967, Zerbini quickly followed suit, performing his first human transplant on May 25, 1968^[1]^. One of Zerbini’s overarching principles that advanced Brazilian cardiac surgery was his conviction that Brazil needed to develop and produce its own medical technology^[1]^. This commitment not only substantially impacted clinical practice within the country, but led to a host of innovations and profoundly impacted many of his disciples, such as Domingo Braile and Adib Jatene^[1]^. Known for his tenacious work ethic, “Omnia vincit labor” (nothing surpasses work) was Zerbini’s motto, which he shared with his disciples^[1]^.

## ADIB DOMINGOS JATENE

Born in 1929, Adib Jatene improved and advanced congenital and adult cardiac surgery in numerous ways; however, he is most known for attempting the first anatomic repair of transposition of the great arteries in 1975, that became known as the Jatene Procedure. This repair was preceded by exhaustive preparation, including examination of 62 pathologic specimens of this and other lesions housed in the Dante Pazzanese Institute of Cardiology, in São Paulo^[2]^. The patient on whom this repair was first performed was a three-month-old baby with a ventricular septal defect. While the patient ultimately died from renal failure three days after surgery, Jatene was not deterred, stating that the case “convinced me that the operation was feasible”^[3]^. This unwavering determination characterized Jatene, who famously stated that a guiding principle of his life was expressed on a bumper sticker he had seen in Brazil: “I prefer one thousand times the tears of defeat than the shame of not having fought”^[4]^. Jatene’s second attempt at the anatomic repair was performed on a 40-day-old infant who also had a ventricular septal defect; this patient survived and he reported the operation in the Journal of the Brazilian Society of Cardiology, followed by the Journal of Thoracic and Cardiovascular Surgery.

Jatene was raised from humble beginnings and faced numerous challenges. He was born in Xapuri, a town near the rainforest in Western Brazil, as one of four sons of Lebanese immigrants^[2]^. His father, a rubber trader, died of yellow fever when Jatene was just two years old^[2,5]^ and eight years later his mother moved the family to Uberlândia in order to provide better education for her children. While he initially considered an engineering career, he ultimately decided to pursue a medical degree at University of São Paulo, planning to return to Western Brazil and practice preventative medicine. However, while in training, he caught the eye of Euryclides de Jesus Zerbini, and in 1951, Jatene joined Zerbini and his team, who performed the first valvular commissurotomy for a patient with mitral stenosis. His career blossomed from there and included innovations related to cardiopulmonary bypass machine development and construction of oxygenators and heat exchangers that not only supplied Brazil but much of South America^[5]^. Other devices he developed included a Starr-Edwards-type heart valve developed in 1962 (two years after the Starr-Edwards valve), defibrillators, pacemakers, and ventricular assist devices^[5]^. Other surgical accomplishments to note include the first coronary artery bypass grafting using reversed saphenous vein in 1970 and his work on geometric ventricular reconstruction^[6]^.

The success of these endeavors is owed to his extraordinary technical skill as well as careful case selection for his first operations^[2]^. While his love for science throughout his career is clearly evident, he never lost sight of the patients stating that “There is no science without humanism. Scientific development is only conceived if directed towards the wellbeing of people”^[4]^.

## MIGUEL BARBERO MARCIAL

**Fig. 1:**
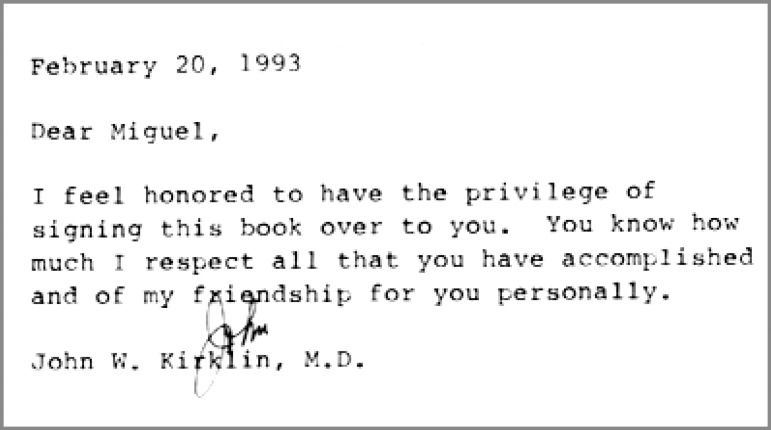
John Kirklin message to Miguel Marcial.

Miguel Barbero Marcial completed his medical degree at University of Litoral Medical School in Rosario, Argentina. After two years of residency in general surgery he was invited by Professor Zerbini to train in cardiovascular surgery in São Paulo, Brazil. Marcial finished his training at the Instituto do Coração (Incor) in 1968, and joined Professor Zerbini’s staff, starting his academic career at University of São Paulo Medical School. During the 1970s, he received a grant to visit congenital cardiac surgeons in the United States of America and New Zealand. During these travels, he met Dr. John Kirklin in Alabama, who became one of his mentors. Marcial maintained a strong professional relationship with Dr. Kirklin; they frequently brainstormed surgical ideas at the annual meeting of the Congenital Heart Surgeons’ Society in Chicago. Dr. Kirklin held Marcial in high regard and respected him for his many contributions to this specialty (Figure 1). Back in Brazil, Marcial structured the pediatric cardiovascular surgery and transplant program at Incor and he is most internationally recognized for his technique for repair of truncus arteriosus without an extra-anatomic conduit. He also is known for his original repair techniques for pulmonary atresia with ventricular septal defect, left ventricular apical approach for the treatment of congenital mitral stenosis, intraventricular repair of double outlet right ventricle with non-committed ventricular septal defect with multiple patches, and cavopulmonary anastomosis excluding portal venous return for protein-losing enteropathy. He also made history by performing the first neonatal heart transplant in South America, which was performed in Brazil, in 1992^[7]^. His development of a classification system for pulmonary atresia with ventricular septal defect is widely used.

## JOSÉ PEDRO DA SILVA

José Pedro da Silva completed his medical school training in Botucatu, São Paulo, and then his general, thoracic, and cardiovascular surgery training in São Paulo before coming to Cleveland Clinic for three years of advanced training and being hired as an associate staff member at the Cleveland Clinic. Dr. da Silva first became interested in the tricuspid valve while working at the Cleveland Clinic in the late 1970s^[8]^. After returning to his homeland in 1981, he remained interested in improving care for patients with Ebstein’s anomaly of the tricuspid valve. After visiting Dr. Alain Carpentier in Paris and observing his monocusp repair technique, da Silva started to develop a repair technique ultimately termed the “cone procedure” or “cone reconstruction”^[8]^. This was an extension of Carpentier’s technique, but it included circumferential mobilization and incorporation of all leaflet tissue, specifically the septal leaflet, not just the anterior and inferior leaflets originally described by Carpentier. After four years of work refining this technique, da Silva started to use this surgical repair routinely for Ebstein’s anomaly^[8]^. Since that time, this nearly anatomic repair technique has spread worldwide as the operation of choice for repair of Ebstein’s anomaly. In his concern for the care of these patients, da Silva particularly wished to come up with a strategy that would allow female patients with Ebstein’s anomaly to safely bear children, which was previously considered too risky^[8]^. It gives da Silva great pleasure, therefore, that the patient on whom he performed his first cone procedure in 1993, at the age of 12 years, is now in her thirties with two healthy children^[8]^. Throughout the years, da Silva continues to refine his surgical technique and shares his “lessons learned” with surgeons around the world, aiding numerous patients.

The field of congenital cardiac surgery owes a debt of gratitude to Brazil for its many important contributions to the field of congenital heart surgery. The courageous, innovative, and gritty style of the Brazilian cardiac surgeons overcame many challenges, but undaunted and with a motivated spirit characteristic of the country, they forged ahead and advanced the field helping innumerable patients around the world.
